# Proposal of a T3 Subclassification for Colon Carcinoma

**DOI:** 10.3390/cancers14246186

**Published:** 2022-12-14

**Authors:** Susanne Merkel, Maximilian Brunner, Carol-Immanuel Geppert, Robert Grützmann, Klaus Weber, Abbas Agaimy

**Affiliations:** 1 Department of Surgery, Friedrich-Alexander-Universität Erlangen-Nürnberg, 91054 Erlangen, Germany; 2 Comprehensive Cancer Center Erlangen-European Metropolitan Area of Nürnberg (CCC ER-EMN), 91054 Erlangen, Germany; 3 Institute of Pathology, Friedrich-Alexander-Universität Erlangen-Nürnberg, 91054 Erlangen, Germany

**Keywords:** colon carcinoma, pT3, T3 subdivision, distant metastasis, survival, prognosis, prognostic factor, TNM classification

## Abstract

**Simple Summary:**

One of the most important prognostic factors for patients with colon cancer is the anatomical extent at the time of surgery. It is described by the TNM classification, which is the basis for treatment planning. T refers to the extent of the primary tumor. Usually, four T categories are distinguished. T3 describes invasion into the pericolic tissue and is the most frequent category found in colon carcinomas. A subclassification of T3, as we present here in this retrospective study, helps to better predict prognosis and further optimize treatment and therapeutic standards.

**Abstract:**

The TNM classification system is one of the most important factors determining prognosis for cancer patients. In colorectal cancer, the T category reflects the depth of tumor invasion. T3 is defined by a tumor that invades through the muscularis propria into pericolorectal tissues. The data of 1047 patients with complete mesocolic excision were analyzed. The depth of invasion beyond the outer border of the muscularis propria into the subserosa or into nonperitonealized pericolic tissue was measured and categorized in 655 pT3 patients: pT3a (≤1 mm), pT3b,c (>1–15 mm) and pT3d (>15 mm). The prognosis of these categories was compared. Five-year distant metastasis increased significantly from pT3a (5.7%) over pT3b,c (17.7%) to pT3d (37.2%; *p* = 0.001). There was no difference between pT2 (5.3%) and pT3a or between pT3d and pT4a (42.1%) or pT4b (33.7%). The 5-year disease-free survival decreased significantly from pT3a (77.4%) over pT3b,c (65.4%) to pT3d (50.1%; *p* = 0.015). No significant difference was found between pT2 (80.5%) and pT3a or between pT3d and pT4a (43.9%; *p* = 0.296) or pT4b (53.4%). The prognostic inhomogeneity in pT3 colon carcinoma has been demonstrated. A three-level subdivision of T3 for colon carcinoma in the TNM system into T3a (≤1 mm), T3b (>1–15 mm), and T3c (>15 mm) is recommended.

## 1. Introduction

The TNM classification system [1,2] is one of the most important factors determining treatment and prognosis for patients diagnosed with solid cancer. Advances in diagnostics and treatment require regular optimization of the staging system. The T-category reflects the primary tumor, either defined by tumor size (largest diameter) as in many organs, by the depth of the tumor invasion as in colorectal cancer (CRC), or by combined sets of criteria. In CRC, T3 is defined by a tumor that invades through the muscularis propria into pericolorectal tissues. T4a and T4b tumors penetrate through the visceral peritoneum (T4a) or invade directly or adhere to adjacent organs or structures (T4b).

Prognostic inhomogeneity of the pT3 category has already been shown for rectal carcinoma after primary surgical treatment [3] and after preoperative neoadjuvant chemoradiation followed by surgery [4]. A proposal for a subdivision of the pT3 category was presented for both rectal and colon carcinomas in the various editions of the TNM supplements [5,6,7,8]. However, thus far, it has not been included in the official TNM classification. Here, we present a subclassification of pT3 in colon carcinoma to demonstrate the wide range of prognoses of these tumors. Furthermore, we wanted to show the overlap of pT3 with pT2 at a low invasion depth and the overlap of pT3 with pT4 at an advanced invasion depth.

## 2. Materials and Methods

### 2.1. Inclusion and Exclusion Criteria of the Study

Data from patients with the following inclusion criteria were analyzed: invasive colon carcinoma, no appendix carcinoma; pT-category > pT1; more than 16 cm from the anal verge; treatment by complete mesocolic excision (CME) at the Department of Surgery of the University Hospital Erlangen, Germany, between 1998 and 2015; curative resection (R0 by macroscopic and microscopic examination); no neoadjuvant treatment; no distant metastases at diagnosis; carcinoma not arising in the setting of familial adenomatous polyposis, ulcerative colitis or Crohn’s disease. Thirty-four of 1081 patients (3.1%) had to be excluded: 22 patients because of missing data on the depth of invasion into subserosa or into nonperitonealized pericolic tissue and 12 patients because of missing follow-up information. In summary, data from 1047 consecutive patients were analyzed.

### 2.2. Description of the pT3 Subdivision

**Figure 1:**
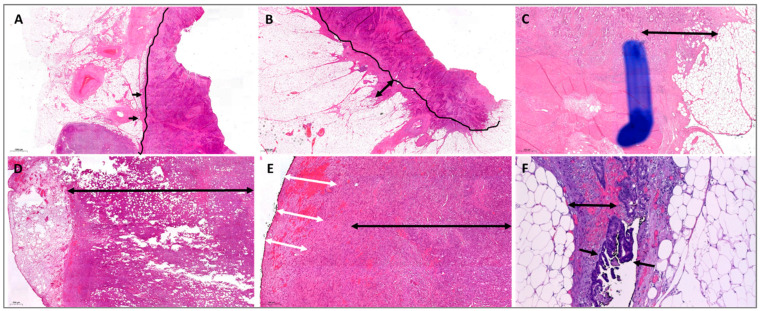
Histological presentation of the categories pT3a-d and pT4 in colorectal carcinoma. Double-headed black arrows highlight the depth of the subserosal invasion; arrows highlight neoplastic cells. (**A**): CRC completely obliterating the lamina muscularis propria (black line) and showing early involvement of the subserosa (arrows), indicating pT3a. (**B**): Similar illustration as in (**A**), but with 4 mm subserosal extension indicated by the double-head arrow (pT3b). (**C**): This case showed subserosal invasion >5 but <15 mm, corresponding to pT3c. (**D**): Example of pT3d showing extensive (>15 mm) subserosal invasion covered by edematous but tumor-free subserosa on the left. (**E**): This case formally qualifies as pT3d. However, the residual subserosa was replaced by a hemorrhagic granulating inflammatory reaction (double-headed white arrows), so sealed serosal penetration cannot be reliably ruled out in such cases. (**F**): Classic pT4 status showing neoplastic tissue on top of the serosa (arrows). However, a hemorrhagic granulating inflammatory reaction is seen (double head arrow), resulting in partially sealed serosal penetration (such cases might later seal completely and hence mimic the case shown in (**E**)).

In pT3 carcinomas, the depth of invasion beyond the outer border of the muscularis propria into the subserosa or into nonperitonealized pericolic tissue was measured and categorized by the pathologist into four groups: pT3a, ≤1 mm; pT3b, >1–5 mm; pT3c, >5–15 mm; pT3d, >15 mm (Figure 1); then, the two intermediate subgroups pT3b and pT3c were combined into a single subgroup for statistical analysis. Here, we distinguish between the three categories: pT3a (invasion up to 1 mm), pT3b,c (invasion more than 1 mm up to 15 mm), and pT3d (invasion more than 15 mm).

### 2.3. Tumor Documentation

Epidemiological data, treatment, histopathological findings, and follow-up data were collected prospectively at the Erlangen Registry for Colorectal Carcinomas (ERCRC). The detailed documentation of the histopathological examinations allowed the classification of all carcinomas in accordance with the 8th edition of the UICC TNM classification [2].

According to its embryologic origin, the right colon was defined from the cecum to the proximal two-thirds of the transverse colon; the left colon extended from the distal third of the transverse colon to the sigmoid colon. 

### 2.4. Surgical Procedure, Adjuvant Treatment, and Follow-Up

Complete mesocolic excision (CME) [9] was introduced and developed in 1985 and has consequently been implemented since 1995 [10]. With the exception of 11 patients who underwent laparoscopic surgery, all patients were operated on by an open approach. The median number of regional lymph nodes that were examined in the specimens was 29 (range 8–145). In 1041 of 1047 patients (99.4%), twelve or more lymph nodes were examined.

Adjuvant treatment was administered in 244 of 373 patients (65.4%) with stage III disease and in 13 of 472 patients (2.8%) with high-risk stage II disease, mostly using 5-fluorouracil and folinic acid with or without oxaliplatin according to the evidence-based German guideline for colorectal cancer that was valid at the time of treatment [11].

Patients were followed up for at least 5 years with physical examination, estimation of carcinoembryonic antigen (CEA) levels, abdominoperineal ultrasonography, chest X-ray, and colonoscopy. Thereafter, vital status was checked annually. 

### 2.5. Statistical Analysis

The chi-square test and Fisher’s exact test were used to compare categorical data, and the Mann–Whitney U test was used to compare continuous data. The Kaplan–Meier method was applied to analyze the rates of distant metastases, disease-free survival, overall survival, and cancer-related survival. For the analysis of disease-free survival, the first occurrence of locoregional or distant recurrence or death from any cause was defined as an event. For estimation of overall survival, we defined death from any cause as an event. For the analysis of cancer-related survival, an event was defined as death from colon cancer, either because of recurrence (locoregional or distant) or because of postoperative death following reoperation. The 95% confidence intervals (CIs) were calculated according to the method described by Greenwood [12]. The survival curves were compared using a log-rank test. Cox regression analysis was used for multivariate analyses and was adjusted for age in survival analyses. For the identification of independent prognostic factors, all variables with *p* < 0.05 in univariate analysis were included in the multivariate model. A *p*-value < 0.05 was considered significant. All analyses were performed using the statistical software package SPSS^®^ version 24.0 (IBM, Armonk, New York, NY, USA).

## 3. Results

### 3.1. Patient Characteristics

The demographic and clinicopathological characteristics of the 1047 patients are shown in Table 1. A total of 655 patients were classified as pT3 and divided into subgroups pT3a, n = 155 (23.7%); pT3b,c, n = 433 (66.1%); and pT3d, n = 67 (10.2%). Table 2 presents the distribution of typical prognostic factors. We found significant differences in the distribution of prognostic factors between pT3a, pT3b,c, and pT3d carcinomas. High-grade carcinomas and those with lymphatic invasion were found to be significantly less frequent in pT3a than in pT3b,c (*p* = 0.016 and *p* < 0.001). Lymph node-positive carcinomas and those with lymphatic and/or venous invasion were found significantly more frequently in pT3d than in pT3b,c carcinomas (*p* < 0.001, *p* = 0.001, and *p* = 0.006). At the same time, pT3a carcinomas showed a similar distribution of these prognostic factors as pT2 carcinomas, and pT3d carcinomas had a similar distribution as pT4a and pT4b carcinomas. No differences were identified with respect to the location of the tumors within the right or left colon.

The median follow-up of all patients was 8 years (range 0–22 years). During follow-up, locoregional recurrences were observed in 27 patients (2.6%), and distant metastases were observed in 173 patients (16.5%). At the time of analysis, 512 patients (48.9%) had died: 39 (3.7%) postoperatively, 139 (13.3%) related to recurrent disease, 62 (5.9%) from other malignancies, and 272 (26.0%) due to other nonmalignant diseases. 

### 3.2. Locoregional Recurrences

The 5-year rate of locoregional recurrence for all patients was 2.9% (95% CI 1.7–4.1%; Table S1).

### 3.3. Distant Metastases

**Figure 2:**
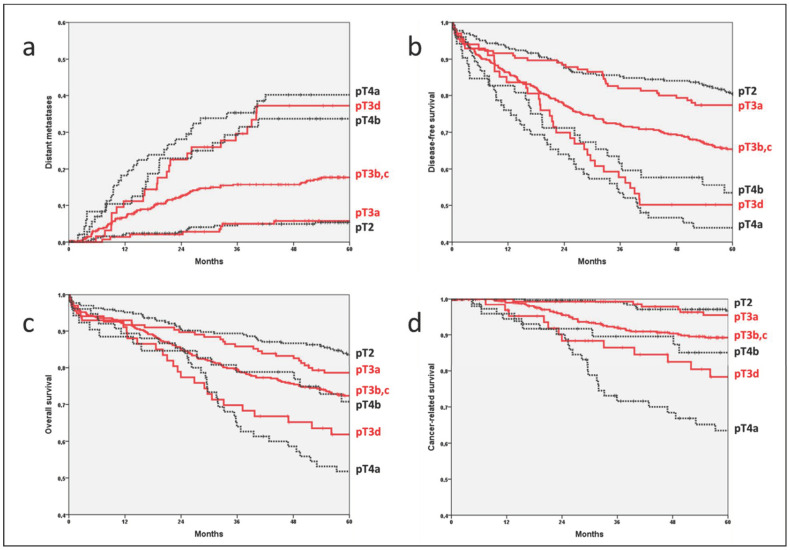
Comparison of the prognosis between patients with pT2 (n = 265), pT3a (n = 155) and pT3b,c (n = 433) and pT3d (n = 67), pT4a (n = 75) and pT4b (n = 52) colon carcinomas: (**a**) distant metastases, (**b**) disease-free survival, (**c**) overall survival, (**d**) cancer-related survival.

The 5-year rate of distant metastases for all patients was 16.4% (95% CI 14.0–18.8%). Distant metastases increased significantly from pT3a over pT3b,c to pT3d, i.e., from 5.7% via 17.7% to 37.2% (*p* = 0.002 and 0.001; Table 3a, Figure 2a) within 5 years. At the same time, there was no difference between pT2 and pT3a with 5.3% and 5.7% (*p* = 0.993) or between pT3d and pT4a and pT4b with 37.2%, 42.1% and 33.7% (*p* = 0.579 and *p* = 0.403). In patients without regional lymph node metastases (pN0), a significant difference in the frequency of distant metastasis was identified between pT3a and pT3b,c (4.1% vs. 13.0%; *p* = 0.011), which could be confirmed in multivariate analysis (Table S2). In contrast, in lymph node-positive patients (pN1,2), a significant difference was found between pT3b,c and pT3d (26.1% vs. 56.2%; *p* = 0.001).

Thirty patients developed peritoneal metastases, 20 of whom had distant metastases in other locations. This was observed extremely rarely in pT2 and pT3a patients (1/265 and 1/155) and was rare in pT3b,c and pT3d patients (12/433 and 1/67). pT4a patients were diagnosed with peritoneal metastases much more frequently (12/75; 16%), followed by pT4b patients (3/52; 6%). 

### 3.4. Disease-Free Survival

A 5-year disease-free survival rate of 67.9% (65.2–70.6%) was observed for all patients. The differences between the pT categories were similar to those for distant metastasis. The 5-year disease-free survival decreased significantly from pT3a (77.4%) over pT3b,c (65.4%) to pT3d (50.1%; *p* = 0.015 and 0.033; Table 3b, Figure 2b). No significant difference was found between pT2 (80.5%) and pT3a (77.4%; *p* = 0.844) or between pT3d (50.1%) and pT4a (43.9%; *p* = 0.296) and pT4b (53.4%; *p* = 0.177). In pN0 patients, a significantly better 5-year disease-free survival was found in pT3a (80.2%) compared to pT3b,c (68.7%; *p* = 0.012), while in pN1,2 patients, it was significantly better in pT3b,c (59.9%) compared to pT3d (34.2%; *p* = 0.007).

### 3.5. Overall Survival

The 5-year overall survival rate was 73.9% (71.2–76.6%) for all patients. The overall survival rates decreased from pT3a (78.6%) over pT3b,c (72.4%) to pT3d (61.9%; Table 4a, Figure 2c). However, the significance level was not reached. Only in pN1,2 patients was there a significant decrease in the 5-year rate from 68.6% in pT3b,c to 48.9% in pT3d carcinomas (*p* = 0.011).

### 3.6. Cancer-Related Survival

Finally, the 5-year rate of cancer-related survival of all patients was 89.3% (87.3–91.3%). It decreased significantly from 95.5% in pT3a patients over 89.2% in pT3b,c (*p* = 0.025) to 78.3% in pT3d (*p* = 0.039; Table 4b, Figure 2d). Again, a significant difference in pN1,2 patients was found between pT3c,d and pT3d (82.8% vs. 61.8%; *p* = 0.004).

In nearly all the analyses, a nonsignificant rather worse prognosis was observed in pT4a patients than in pT4b patients, possibly due to the higher rate of metachronous peritoneal metastases in pT4a carcinomas.

### 3.7. Cox Regression Analysis

In univariate and multivariate Cox regression analyses (Table 5 and Table 6), pT3b,c was defined as the reference group and set as 1.0. This enabled us to investigate whether the prognosis of pT3a is significantly better and the prognosis of pT3d patients is significantly worse compared to pT3b,c. In the multivariate analysis of distant metastasis, we found that metastases were diagnosed significantly less frequently in pT3a carcinomas than in pT3b,c, while they occurred almost significantly more frequently in pT3d carcinomas. In multivariate analysis of disease-free survival, the prognosis of pT3a patients was found to be significantly better, and the prognosis of pT3d patients was nonsignificantly worse when compared to pT3b,c.

### 3.8. Adjuvant Chemotherapy

In Stage II, 7 of 408 patients (1.7%) received adjuvant chemotherapy (pT3a: n = 1/106, pT3b,c: n = 5/273, pT3d: n = 1/28). None of these patients with adjuvant chemotherapy developed distant metastases. One patient died within five years from distant metastases of an unknown primary.

In stage III, 161 of 247 patients (65.2%) received adjuvant chemotherapy (pT3a: n = 30/49, pT3b,c: n = 108/160, pT3d: n = 23/38). The 5-year rates of distant metastases were 39.7% (95% CI 27.4–52.0) in stage III patients who did not receive chemotherapy and 22.4% (15.9–28.9) in patients who underwent adjuvant chemotherapy (*p* = 0.003). In the 86 patients with stage III who did not receive adjuvant chemotherapy, the 5-year rates of distant metastases were as follows: pT3a (n = 19) 7.1%; pT3b,c (n = 52) 10.2%; pT3d (n = 15) 87.2%; pT3a vs. pT3b,c: *p* = 0.151; pT3b,c vs. pT3d: *p* = 0.051; pT3a vs. pT3d: *p* < 0.001. In patients with stage III disease who underwent adjuvant chemotherapy, the 5-year rates of distant metastases also increased with the depth of invasion: pT3a (n = 30) 10.2%; pT3b,c (n = 108) 21.2%; pT3d (n = 23) 44.1%; pT3a vs. pT3b,c: *p* = 0.171; pT3b,c vs. pT3d; *p* = 0.024; pT3a vs. pT3d: *p* = 0.003. Further details on the prognosis for pT3 subclassification in stage III patients with and without adjuvant chemotherapy are presented in Table S3.

## 4. Discussion

The depth of the invasion beyond the muscularis propria is an important prognostic factor in colon carcinoma. The TNM classification system classifies carcinomas that invade the pericolic fat tissue as pT3. In contrast, carcinomas that already have involved the serosa or adjacent organs or structures are classified as pT4, more precisely, pT4a and pT4b, respectively. The TNM system does not provide a subclassification for pT3. The prognostic inhomogeneity of pT3 and ypT3 has been discussed in previous studies for rectal carcinomas [3,4]. Our analyses also show that there is a wide range of prognoses in colon carcinomas depending on the depth of infiltration into the pericolic fat.

In all resected pT3 specimens, tumor invasion beyond the muscularis propria into the pericolic fat was measured in mm and transformed to an ordinal scale. Initially, during data collection, we used a four-level scale of pT3a, b, c, and d. Different from the analysis of rectal carcinoma, where we proposed a subdivision of up to 5 mm and more than 5 mm, we found in colon carcinomas that pT3b (invasion of >1–5 mm) and pT3c (>5–15 mm) had a very similar prognosis. Therefore, we suggest a three-level subdivision of pT3 for colon carcinomas into pT3a (≤1 mm), pT3b,c (>1–15 mm), and pT3d (>15 mm).

The majority of patients, approximately two-thirds, belong to the intermediate risk group (pT3b,c) with a depth of invasion of more than 1 mm but not more than 15 mm. However, patients with a minimal invasion of up to 1 mm (pT3a) have a favorable prognosis that is comparable to patients with pT2 carcinomas. This is the case for 26% of pT3 patients without lymph node metastases and for almost 21% of pT3 patients with lymph node metastases. In contrast, patients with tumor invasion into the pericolic fat tissue of more than 15 mm (pT3d) have a significantly worse prognosis, comparable to patients with pT4 carcinomas. This concerns 7% of pT3 pN0 patients and 12% of pT3 pN1,2 patients.

The inhomogeneity of pT3 could be confirmed for stage III patients without and with adjuvant chemotherapy. The 5-year rate of distant metastases increased with the depth of invasion in the group of patients without adjuvant chemotherapy and in the patients with adjuvant treatment. Patients with pT3d pN1,2 carcinomas without adjuvant chemotherapy had the worst prognosis, with a 5-year rate of distant metastasis of 87.2% and a 5-year rate of disease-free survival of only 6.7%. Between 1998 and 2015, adjuvant chemotherapy regimens evolved from 5-FU/FS to combinations with oxaliplatin, such as FOLFOX or XELOX. The different chemotherapy regimens were not included in the analyses. Currently, the chemotherapy regimen is selected primarily with regard to the age and comorbidities of the patients. Whether different regimens can be recommended for the different pT3 subcategories will be an important future question.

Recently, Panarelli et al. [13] highlighted the lack of consistent reproducibility of the AJCC/UICC criteria for classifying deeply invasive colon cancers, in particular, the distinction between deep pT3 (comparable to pT3d) and pT4a (invasion of the serosa). In general, moderate agreement (κ= 0.52) was achieved by gastrointestinal pathologists when the tumor had a well-delineated pushing deep border. Still, it was only slight (κ= 0.16) when an inflammatory reaction was present at the advancing tumor edge. The problems with assigning deep T3 versus T4a status reflect the ambiguous definition of serosal penetration as a defining feature of pT4a. In our own experience and as highlighted in the aforementioned Panarelli et al.’s study, this issue is complicated by several factors, including limited reliability on gross findings that are considered suspicious for serosal penetration and the degree of sampling for its verification. On occasion, grossly suspected serosal penetration turns out to be just a deep T3 with an associated inflammatory reaction at the advancing deep tumor edge. Diffusely infiltrating carcinomas are frequently associated with fibroinflammatory and fibrovascular granulating tissues that may result in complete obliteration of the residual subserosal tissue at the advancing tumor edge. This issue has been highlighted in the study by Panarelli et al. as one of the major confusing factors in assigning a pT3 versus pT4a category. Another confounding factor is the tendency of inured or preached serosal tissue to undergo a process of healing, which ultimately results in an apparently intact fibroinflammatory layer between the advancing tumor edge and the serosal surface. This finding might justify assigning a T3 instead of T4a category by general surgical pathologists. Adherence of adjacent omental, mesenteric, or other peritoneal fatty tissue may seal such foci of serosal penetration, suggesting pT3. In their study, Panarelli et al. concluded that the histologic criteria for recognizing serosal penetration represent a persistent source of diagnostic ambiguity for both gastrointestinal and general surgical pathologists in assigning the pT category for colon carcinomas. This significant overlap and confusion regarding deep pT3 versus pT4 could explain the very similar prognosis of the two categories observed in our current study.

The most important difference in the treatment between pN0 and pN+ colon cancer patients is that adjuvant chemotherapy is generally recommended for stage III (pN+) patients. In stage II (pN0), adjuvant chemotherapy is limited to high-risk groups. Therefore, identifying high-risk and low-risk groups is particularly important in stage II. pT3d cancers mainly behave like pT4 cancers. Therefore, these patients belong to a high-risk group for whom adjuvant therapy should also be discussed in stage II [14].

Another risk factor that plays an important role in prognosis, especially in node-negative colon cancer, is the number of regional lymph nodes examined [15,16]. However, in the cohort that we analyzed, only six of 1047 patients had fewer than 12 lymph nodes examined. Swanson et al. also found the left colon to be a risk factor in stage II colon cancer [16]. This could not be confirmed by our data. However, in a previous analysis, including patients treated between 1981 and 1997 at our department, we identified left-sided carcinomas of the sigmoid or descending colon, emergency presentation, a depth of invasion of >15 mm beyond the outer border of the muscularis propria and pT4 lesions as the major risk factors for stage II colon carcinoma [14]. The current German S3 guideline for colorectal carcinoma recommends considering adjuvant chemotherapy in stage II patients with selected risk situations (pT4, tumor perforation, emergency presentation, <12 regional lymph nodes examined). In cases of proven microsatellite instability (MSI-H), adjuvant chemotherapy should not be applied in stage II. This is based on the better long-term prognosis of patients with MSI-H colon carcinoma [17].

The distribution of prognostic factors in the different subcategories of pT3 colon carcinomas showed an increasing rate of lymph node metastases, high-grade carcinomas, lymphatic invasion, and venous invasion with increasing depth of invasion. The attempt to present the subcategories of pT3 as an independent prognostic factor in multivariate Cox regression analysis has been successful only with limitations, most likely for distant metastasis. However, multivariate Cox regression analyses may represent a certain over-adjustment in this case. Nevertheless, we can prove for distant metastasis that if we set pT3b,c to 1.0, distant metastasis in pT3a and pT2 is similarly less frequent, and distant metastasis in pT3d and pT4a,b is similarly more frequent. The hazard ratios and their confidence intervals are similar in both cases. For disease-free survival, this could be shown less clearly.

To our knowledge, this is the only published study that examines a subdivision of pT3 in colon carcinoma patients. Further studies are therefore encouraged to confirm our results. In addition to the different treatment methods for colon and rectal carcinomas, the differences in the optimized subclassifications for colon (pT3a ≤ 1 mm; pT3b,c > 1–15 mm; pT3d > 15 mm) and rectal (pT3a,b ≤ 5 mm; pT3c,d > 5 mm) [3,4] carcinomas are one more reason to separate the TNM classification for colon and rectal carcinomas.

Our study has some limitations regarding the thickness of pericolic fat tissue. Usually, the pericolic fat is thinner in slim people than in overweight patients. Consequently, there could be subgroups of patients for whom the subclassification may be less meaningful. To the best of our knowledge, there is no study on the distribution patterns of pericolic fatty tissue, e.g., depending on age, sex, or body mass index. For low rectal cancer, Wong et al. [18] examined the thickness of mesorectal fat in 25 Chinese patients with T3 rectal carcinoma. They found the lateral mesorectal fat on the left and right sides to be thicker than the anterior or posterior. The mean thickness at 10 cm from the anal verge was <5 mm in 71% and <15 mm in 95% of the Chinese patients. Allen et al. [19] found a strong correlation between the volume of the visceral compartment area and the mesorectal area in both sexes but not for body mass index. Further limitations of this study are the long study duration with changes in adjuvant treatment over time, the retrospective character, and the single-center analysis.

## 5. Conclusions

**Figure 3:**
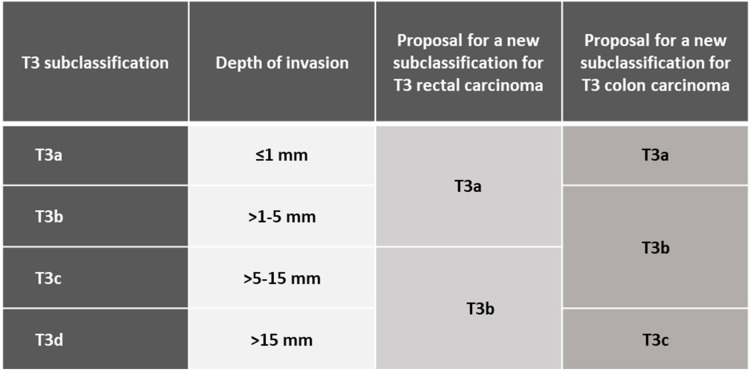
Proposal for a new subclassification for T3 rectal and colon carcinoma.

The depth of the invasion beyond the muscularis propria is an important independent prognostic factor in pT3 colon carcinoma. A three-level subdivision of T3 in the TNM system into T3a (≤1 mm), T3b (>1–15 mm), and T3c (>15 mm) is recommended (Figure 3).

## Figures and Tables

**Table 1:** Patient and tumor characteristics for 1047 patients.

		n	(%)
Age median (range) (years)		69 (21–99)	
Sex	Male	602	57.5
	Female	445	42.5
ASA *	ASA I–II	730	70.9
	ASA III–IV	300	28.7
Tumor site	Right colon	511	48.8
	Left colon	536	51.2
Emergencies	Elective surgery	931	88.9
	Emergency presentation	116	11.1
Surgical procedure	Colon standard resection	787	75.2
	Colon extended resection	260	24.8
Multivisceral resection	No	928	88.6
	Yes	119	11.4
Adjuvant treatment	No	790	75.5
	Yes	257	24.5
pT category	pT2 (muscularis propria)	265	25.3
	pT3a (≤1 mm)	155	14.8
	pT3b,c (>1–15 mm)	433	41.4
	pT3d (>15 mm)	67	6.4
	pT4a (serosa)	75	7.2
	pT4b (other organs)	52	5.0
pN category	pN0	674	64.4
	pN1	261	24.9
	pN2	112	10.7
Grading	G1,2	699	66.8
	G3,4	348	33.2
Lymphatic invasion	L0	737	70.5
	L1	309	29.5
Venous invasion	V0	1012	96.7
	V1	34	3.3

*ASA* American Society of Anesthesiologists Classification; * ASA missing in 17 patients.

**Table 2:** Distribution of prognostic factors (n = 1047).

	pT2	*p*	pT3a (≤1 mm)	*p*	pT3b,c (>1–15 mm)	*p*	pT3d (>15 mm)	*p*	pT4a	*p*	pT4b	*p*_overall_
n	265		155		433		67		75		52	
	n (%)		n (%)		n (%)		n (%)		n (%)		n (%)	
Right colon	131 (49.4)		79 (51.0)		211 (48.7)		28 (42)		38 (51)		24 (46)	
Left colon	134 (50.6)	0.564	76 (49.0)	0.632	222 (51.3)	0.290	39 (58)	0.290	37 (49)	0.617	28 (54)	0.862
pN0	202 (76.2)		106 (68.4)		273 (63.0)		29 (43)		34 (45)		30 (58)	
pN1	54 (20.4)		38 (24.5)		110 (25.4)		19 (28)		21 (28)		19 (37)	
pN2	9 (3.4)	0.175	11 (7.1)	0.257	50 (11.5)	<0.001	19 (28)	0.965	20 (27)	0.011	3 (6)	<0.001
G1,2	204 (77.0)		115 (74.2)		275 (63.5)		40 (60)		39 (52)		26 (50)	
G3,4	61 (23.0)	0.592	40 (25.8)	0.016	158 (36.5)	0.548	27 (40)	0.356	36 (48)	0.825	26 (50)	<0.001
L0	225 (85.2)		129 (83.2)		285 (65.8)		30 (45)		35 (47)		33 (64)	
L1	39 (14.8)	0.653	26 (16.8)	<0.001	148 (34.2)	0.001	37 (55)	0.821	40 (53)	0.062	19 (37)	<0.001
V0	262 (99.2)		153 (98.7)		419 (96.8)		60 (90)		69 (92)		49 (94)	
V1	2 (0.8)	0.587	2 (1.3)	0.202	14 (3.2)	0.006	7 (10)	0.614	6 (8)	0.630	3 (6)	<0.001

**Table 3:** Distant metastases and disease-free survival (n = 1047).

	pT2	*p*	pT3a (≤1 mm)	*p*	pT3b,c (>1–15 mm)	*p*	pT3d (>15 mm)	*p*	pT4a (Serosa)	*p*	pT4b (Other Organs)
**(a) Distant metastases**											
**Any pN**	n = 265		n = 155		n = 433		n = 67		n = 75		n = 52
5-year rate	5.3%	0.993	5.7%	0.002	17.7%	0.001	37.2%	0.579	42.1%	0.403	33.7%
(95% CI)	(2.6–8.0)		(1.8–9.6)		(14.0–21.4)		(24.7–49.7)		(30.3–53.9)		(20.2–47.2)
**pN0**	n = 202		n = 106		n = 273		n = 29		n = 34		n = 30
5-year rate	3.8%	0.936	4.1%	0.011	13.0%	0.951	12.1%	0.263	25.3%	0.212	14.7%
(95% CI)	(1.1–6.5)		(0.2–8.0)		(8.9–17.1)		(0–25.0)		(10.0–40.6)		(1.4–28.0)
**pN1,2**	n = 63		49		160		n = 38		n = 41		n = 22
5-year rate	10.1%	0.803	9.2%	0.069	26.1%	0.001	56.2%	0.855	53.2%	0.623	59.9%
(95% CI)	(2.5–17.7)		(0.6–17.8)		(18.8–33.4)		(39.1–73.3)		(37.1–69.3)		(38.3–81.5)
**(b) Disease-free survival**											
**Any pN**	n = 265		n = 155		n = 433		n = 67		n = 75		n = 52
5-year rate	80.5%	0.844	77.4%	0.015	65.4%	0.033	50.1%	0.296	43.9%	0.177	53.4%
(95% CI)	(75.6–85.4)		(70.7–84.1)		(60.9–69.9)		(37.9–62.3)		(32.7–55.1)		(39.7–67.1)
**pN0**	n = 202		n = 106		n = 273		n = 29		n = 34		n = 30
5-year rate	79.9%	0.161	80.2%	0.012	68.7%	0.619	71.7%	0.226	58.8%	0.157	73.3%
(95% CI)	(74.4–85.4)		(72.6–87.8)		(63.2–74.2)		(55.0–88.4)		(42.3–75.3)		(57.4–89.2)
**pN1,2**	n = 63		49		160		n = 38		n = 41		n = 22
5-year rate	82.3%	0.054	71.4%	0.508	59.9%	0.007	34.2%	0.667	31.7%	0.805	26.0%
(95% CI)	(72.9–91.7)		(58.7–84.1)		(52.3–67.5)		(19.1–49.3)		(17.4–46.0)		(7.2–44.8)

**Table 4:** Overall survival and cancer-related survival (n = 1047).

	pT2	*p*	pT3a (≤1 mm)	*p*	pT3b,c (>1–15 mm)	*p*	pT3d (>15 mm)	*p*	pT4a (Serosa)	*p*	pT4b (Other Organs)
**(a) Overall survival**											
**Any pN**	n = 265		n = 155		n = 433		n = 67		n = 75		n = 52
5-year rate	83.5%	0.634	78.6%	0.119	72.4%	0.249	61.9%	0.108	51.8%	0.068	70.7%
(95% CI)	(79.0–88.0)		(72.1–85.1)		(68.1–76.7)		(50.1–73.7)		(40.4–63.2)		(58.2–83.2)
**pN0**	n = 202		n = 106		n = 273		n = 29		n = 34		n = 30
5-year rate	82.4%	0.300	81.1%	0.058	74.6%	0.162	78.8%	0.058	70.5%	0.182	83.3%
(95% CI)	(77.1–87.7)		(73.7–88.5)		(69.5–79.7)		(63.7–93.9)		(55.2–85.8)		(70.0–96.6)
**pN1,2**	n = 63		49		160		n = 38		n = 41		n = 22
5-year rate	87.1%	0.006	73.5%	0.988	68.6%	0.011	48.9%	0.480	36.6%	0.459	53.6%
(95% CI)	(79.0–95.3)		(61.2–85.8)		(61.3–75.9)		(32.6–65.2)		(21.9–51.3)		(32.4–74.8)
**(b) Cancer-related survival**											
Any pN	n = 265		n = 155		n = 433		n = 67		n = 75		n = 52
5-year rate	96.6%	0.300	95.5%	0.025	89.2%	0.039	78.3%	0.049	63.5%	0.042	85.1%
(95% CI)	(94.2–99.0)		(92.0–99.0)		(86.1–92.3)		(67.3–89.3)		(51.9–75.1)		(74.9–95.3)
**pN0**	n = 202		n = 106		n = 273		n = 29		n = 34		n = 30
5-year rate	97.7%	0.541	96.7%	0.071	92,9	0.087	100%	0.006	84.0%	0.071	96.2%
(95% CI)	(95.5–99.9)		(93.0–100)		(89.6–96.2)				(71.1–96.9)		(88.8–100)
**pN1,2**	n = 63		49		160		n = 38		n = 41		n = 22
5-year rate	93.2%	0.565	92.8%	0.217	82.8	0.004	61.8%	0.287	46.5%	0.428	69.6%
(95% CI)	(86.7–99.7)		(85.0–100)		(76.5–89.1)		(44.4–79.2)		(28.5–64.5)		(49.2–90.0)

**Table 5:** Distant metastases, multivariate Cox regression analysis (n = 1047).

			Univariate Analysis			Multivariate Analysis		
		n	Hazard Ratio	95% CI	*p*	Hazard Ratio	95% CI	*p*
Sex	Male	602	1.0					
	Female	445	0.8	0.6–1.1	0.126			
ASA*	ASA I-II	730	1.0					
	ASA III-IV	300	1.3	0.9–1.8	0.148			
Tumor site	Right colon	511	1.0					
	Left colon	536	1.3	1.0–1.8	0.082			
Emergencies	Elective surgery	931	1.0			1.0		
	Emergency presentation	116	2.5	1.7–3.6	<0.001	1.8	1.2–2.7	0.003
Surgical procedure	Colon standard resection	787	1.0					
	Colon extended resection	260	1.2	0.9–1.7	0.308			
pT category	pT2 (muscularis propria)	265	0.4	0.2–0.6	<0.001	0.5	0.3–0.8	0.004
	pT3a (≤1 mm)	155	0.4	0.2–0.7	0.002	0.4	0.2–0.8	0.007
	pT3b,c (>1–15 mm)	433	1.0			1.0		
	pT3d (>15 mm)	67	2.2	1.4–3.6	0.001	1.6	1.0–2.5	0.074
	pT4a (serosa)	75	2.6	1.7–3.9	<0.001	2.1	1.4–3.2	0.001
	pT4b (other organs)	52	2.0	1.1–3.4	0.015	2.0	1.1–3.4	0.017
pN category	pN0	674	1.0			1.0		
	pN1	261	2.3	1.6–3.3	<0.001	2.0	1.4–2.9	<0.001
	pN2	112	5.2	3.6–7.6	<0.001	3.4	2.2–5.3	<0.001
Grading	G1,2	699	1.0			1.0		
	G3,4	348	1.4	1.0–1.9	0.031	1.0	0.7–1.3	0.831
Lymphatic invasion	No	737	1.0			1.0		
	Yes	309	2.7	2.0–3.7	<0.001	1.3	0.9–1.8	0.197
Venous invasion	No	1012	1.0			1.0		
	Yes	34	2.3	1.3–4.3	0.007	1.2	0.7–2.3	0.513

**Table 6:** Disease-free survival, multivariate Cox regression analysis (n = 1047).

			Univariate Analysis			Multivariate Analysis Adjusted for Age		
		n	Hazard Ratio	95% CI	*p*	Hazard Ratio	95% CI	*p*
Sex	Male	602	1.0					
	Female	445	0.9	0.7–1.0	0.072			
ASA*	ASA I-II	730	1.0			1.0		
	ASA III-IV	300	2.4	2.0–2.9	<0.001	1.7	1.4–2.0	<0.001
Tumor site	Right colon	511	1.0					
	Left colon	536	0.9	0.8–1.1	0.265			
Emergencies	Elective surgery	931	1.0			1.0		
	Emergency presentation	116	2.2	1.7–2.7	<0.001	1.5	1.2–1.9	0.002
Surgical procedure	Colon standard resection	787	1.0					
	Colon extended resection	260	1.0	0.9–1.3	0.649			
pT category	pT2 (muscularis propria)	265	0.7	0.6–0.9	0.007	0.8	0.7–1.0	0.096
	pT3a (≤1 mm)	155	0.7	0.5–0.9	0.015	0.7	0.5–1.0	0.024
	pT3b,c (>1–15 mm)	433	1.0			1.0		
	pT3d (>15 mm)	67	1.5	1.1–2.1	0.020	1.2	0.9–1.7	0.281
	pT4a (serosa)	75	1.8	1.4–2.4	<0.001	1.4	1.1–2.0	0.018
	pT4b (other organs)	52	1.4	0.9–1.9	0.095	1.2	0.8–1.7	0.365
pN category	pN0	674	1.0			1.0		
	pN1	261	1.1	0.9–1.4	0.175	1.1	0.9–1.4	0.255
	pN2	112	2.1	1.6–2.7	<0.001	1.6	1.2–2.2	0.001
Grading	G1,2	699	1.0					
	G3,4	348	1.1	0.9–1.3	0.208			
Lymphatic invasion	No	737	1.0			1.0		
	Yes	309	1.5	1.3–1.8	<0.001	1.2	1.0–1.5	0.051
Venous invasion	No	1012	1.0			1.0		
	Yes	34	1.7	1.2–2.6	0.008	1.3	0.8–2.0	0.251

## Data Availability

The data presented in the study are available on request from the corresponding author with the permission of the Institutional Research Ethics Committee of Friedrich-Alexander Universität Erlangen-Nürnberg, Germany.

## Supplementary Materials

The following supporting information can be downloaded at: https://www.mdpi.com/article/10.3390/cancers14246186/s1, Table S1: Locoregional recurrences (n = 1047); 5-year rate all patients 2.9% (1.7–4.1%); Table S2: Overall survival, multivariate Cox regression analysis; Table S3: Prognosis in patients with stage III with and without adjuvant chemotherapy (n = 247).
