# Supplementary material for: Proposal of a T3 Subclassification for Colon Carcinoma
Source: Cancers (Basel). 2022 Dec 14;14(24):6186. doi: 10.3390/cancers14246186 (PMC9776825; doi:10.3390/cancers14246186)
Supplement: Supplementary file 1 [file cancers-14-06186-s001.zip › cancers-2051890-supplementary.pdf]

Table S1. Locoregional recurrences (n=1047); 5-year rate all patients 2.9% (1.7-4.1%)

	**pT2**	**p**	**pT3a (≤1mm)**	**p**	**pT3b,c (>1-15mm)**	**p**	**pT3d (>15mm)**	**p**	**pT4a (serosa)**	**p**	**pT4b (other organs)**
**a) Locoregional recurrences**											
**Any pN**<br>5-year rate<br>(SE)	n=265<br>1.3%<br>(0.7)	0.626	n=155<br>0.7%<br>(0.7)	0.434	n=433<br>1.7%<br>(0.7)	0.007	n=67<br>7.4%<br>(3.6)	0.100	n=75<br>16.2%<br>(4.8)	0.040	n=52<br>4.7%<br>(3.3)
**pN0**<br>5-year rate<br>(SE)	n=202<br>1.1%<br>(0.8)	0.303	n=106<br>0%	0.268	n=273<br>1.3%<br>(0.8)	0.578	n=29<br>0%	0.188	n=34<br>7.1%<br>(4.9)	0.614	n=30<br>3.6%<br>(3.5)
**pN1,2**<br>5-year rate<br>(SE)	n=63<br>1.7%<br>(1.7)	0.835	49<br>2.4%<br>(2.4)	0.996	160<br>2.3%<br>(1.3)	0.006	n=38<br>13.7%<br>(6.4)	0.170	n=41<br>25.3%<br>(7.9)	0.074	n=22<br>6.7%<br>(6.4)

Table S2. Distant metastases, multivariate Cox regression analysis - for pN0 only, n=674

			Univariate analysis			Multivariate analysis		
		n	Hazard ratio	95% CI	p	Hazard ratio	95% CI	p
Sex	Male	384	1.0					
	Female	290	0.7	0.4-1.1	0.107			
ASA*	ASA I-II	474	1.0					
	ASA III-IV	190	1.3	0.8-2.2	0.296			
Tumor site	Right colon	332	1.0			1.0		
	Left colon	342	2.4	1.4-4.0	0.001	2.3	1.4-3.9	0.002
Emergencies	Elective surgery	605	1.0			1.0		
	Emergency presentation	69	2.3	1.2-4.2	0.010	1.6	0.8-3.1	0.155
Surgical procedure	Colon standard resection	505	1.0					
	Colon extended resection	169	0.9	0.5-1.6	0.847			
pT category	pT2 (muscularis propria)	202	0.3	0.2-0.7	0.002	0.3	0.2-0.7	0.003
	pT3a (≤1 mm)	106	0.3	0.1-0.8	0.016	0.3	0.1-0.9	0.026
	pT3b,c (>1-15 mm)	273	1.0			1.0		
	pT3d (>15 mm)	29	1.0	0.4-2.9	0.962	1.9	0.3-2.3	0.707
	pT4a (serosa)	34	2.0	1.0-4.1	0.065	0.8	0.9-3.9	0.107
	pT4b (other organs)	30	0.9	0.3-2.6	0.880	1.6	0.3-2.3	0.698
Grading	G1,2	489	1.0					
	G3,4	185	1.0	0.6-1.7	0.956			
Lymphatic invasion	No	585	1.0					
	Yes	89	1.3	0.7-2.5	0.364			
Venous invasion	No	659	1.0			1.0		
	Yes	15	2.9	1.1-7.9	0.039	1.6	0.5-4.6	0.409

Table S3 Prognosis in patients with stage III with and without adjuvant chemotherapy (n=247)

	pT3a (≤1 mm)	p	pT3b,c (>1-15 mm)	p	pT3d (>15 mm)	p (pT3a vs pT3d)
**Distant metastases**						
**Patients without chemotherapy**	n=19		n=52		n=15	
5-year rate	7.1	0.151	10.2	0.051	87.2	<0.001
(95% CI)	0-20.6		0-21.2		64.3-100	
**Patients with adjuvant chemotherapy**	n=30		n=108		n=23	
5-year rate	10.2	0.171	21.2	0.024	44.1	0.003
(95% CI)	0-21.2		13.4-29.0		23.5-64.7	
**Disease-free survival**						
**Patients without chemotherapy**	n=19		n=52		n=15	
5-year rate	47.4	0.967	30.6	0.178	6.7	0.002
(95% CI)	24.9-69.9		18.1-43.1		0-19.2	
**Patients with adjuvant chemotherapy**	n=30		n=108		n=23	
5-year rate	86.7	0.195	74.0	0.028	52.2	0.002
(95% CI)	74.5-98.9		65.8-82.2		31.8-72.6	
